# How Ships' Surgeons Are Appointed

**Published:** 1911-09-02

**Authors:** 


### HOW SHIPS' SURGEONS ARE APPOINTED.

Many more newly qualified graduates would like to take a voyage or two across the world, before settling down to practice or entering the Services, if they knew with what facility ship appointments may be obtained. A voyage or two will invigorate the student after years of study, and leave behind with him for the rest of his life the pleasantest retrospection.

All the great shipping firms are required by the Board of Trade regulations to carry a surgeon when passengers are aboard and when the crew exceeds a certain number, and it is therefore necessary for the mercantile marine of this country to find a large number of medical men annually in order to comply with the Government regulations. It is generally supposed that the best lines of steamers require an agreement extending over a term of years, but such is not the case, for the Merchant Shipping Act forbids an agreement extending beyond the voyage. Even when a company gets hold of a man who suits its requirements, and wishes to retain his services for an extended period, it is not possible to bind him beyond the voyage out and home. The large lines, such as the Peninsular and Oriental, British India, Orient, and Union-Castle lines, get, of course, the pick of the men, and are able to require better qualifications and to stipulate for higher attainments.

Preference is given on these lines to men who have obtained a diploma in tropical medicine or who have held the post of house surgeon or house physician in a general hospital. The pay varies from £10 to £12 a month, with comfortable quarters and all found. The only initial expense is the uniform, and as most of this is inexpensive and of a kind that can be worn in ordinary life (when the buttons have been changed), this item affects but little the advantages of the service. Although the ship's surgeon is not authorised to charge fees for services rendered, a certain number of fees are paid, especially on the lines trading to Australia and South Africa, and these average from 15 to 25 per cent. in addition to the pay.

Although the conditions on the less important lines are not so good, the requirements are not so exacting, and men who have not taken a high qualification or who have not held a house appointment may make voyages under very comfortable circumstances. Besides seeing the world, life as a ship's surgeon has many attractions, interesting and sometimes valuable friendships are made, and if "the Doctor" is willing to throw himself into the life of the ship, he soon finds himself at home, and as his duties but rarely take him more than two or three hours daily, he has plenty of time to devote to reading or to actively sharing in the many entertainments and amusements that are got up on all the great lines of passenger ships. These vessels carry officers who are the pick of the mercantile marine, and they are equipped in the best and most sumptuous manner. A newly-qualified graduate who obtains an appointment in a liner sees the world under very favourable conditions.

It may be interesting to add two typical examples. Candidates for the position of surgeon in the Orient Company's service must be over twenty-three and under forty years of age, be duly qualified and registered in both medicine and surgery, and undertake to serve at least two voyages if so required by the managers. The pay commences at the rate of £10 per month, and surgeons are authorised to make a small charge to first and second-class saloon passengers for services rendered. The rate of pay on the Union-Castle Line is £10 per month. British India Steam Navigation Company surgeons are carried in the passenger steamers trading between London and Calcutta. Surgeons are engaged for the voyage, which occupies about three months, the pay being £8 per month.

